# Survival Analysis of COPD Patients in a 13-Year Nationwide Cohort Study of the Brazilian National Health System

**DOI:** 10.3389/fdata.2021.788268

**Published:** 2022-02-07

**Authors:** Ludmila Peres Gargano, Isabella de Figueiredo Zuppo, Mariana Martins Gonzaga do Nascimento, Valéria Maria Augusto, Brian Godman, Juliana de Oliveira Costa, Francisco Assis Acúrcio, Juliana Álvares-Teodoro, Augusto Afonso Guerra

**Affiliations:** ^1^Department of Social Pharmacy, Faculty of Pharmacy, Federal University of Minas Gerais, Belo Horizonte, Brazil; ^2^Sistema Único de Saúde (SUS) Collaborating Centre – Technology Assessment & Excellence in Health, Faculty of Pharmacy, Federal University of Minas Gerais, Belo Horizonte, Brazil; ^3^Department of Pharmaceutical Products, Faculty of Pharmacy, Federal University of Minas Gerais, Belo Horizonte, Brazil; ^4^Department of Internal Medicine, School of Medicine, Federal University of Minas Gerais, Belo Horizonte, Brazil; ^5^Department of Pharmacoepidemiology, Strathclyde Institute of Pharmacy and Biomedical Sciences, University of Strathclyde, Glasgow, United Kingdom; ^6^Centre of Medical and Bio-Allied Health Sciences Research, Ajman University, Ajman, United Arab Emirates; ^7^School of Pharmaceutical Sciences, Universiti Sains Malaysia, Penang, Malaysia; ^8^School of Pharmacy, Sefako Makgatho Health Sciences University, Pretoria, South Africa; ^9^Centre for Big Data Research in Health, Faculty of Medicine and Health, The University of New South Wales, Sydney, NSW, Australia

**Keywords:** survival analysis, cohort study, chronic obstructive pulmonary disease (COPD), real world data (RWD), record linkage, Brazil

## Abstract

**Background:**

Chronic obstructive pulmonary disease (COPD) has an appreciable socioeconomical impact in low- and middle-income countries, but most epidemiological data originate from high-income countries. For this reason, it is especially important to understand survival and factors associated with survival in COPD patients in these countries.

**Objective:**

To assess survival of COPD patients in Brazil, to identify risk factors associated with overall survival, including treatment options funded by the Brazilian National Health System (SUS).

**Methodology:**

We built a retrospective cohort study of patients dispensed COPD treatment in SUS, from 2003 to 2015 using a National Database created from the record linkage of administrative databases. We further matched patients 1:1 based on sex, age and year of entry to assess the effect of the medicines on patient survival. We used the Kaplan-Meier method to estimate overall survival of patients, and Cox's model of proportional risks to assess risk factors.

**Result:**

Thirty seven thousand and nine hundred and thirty eight patients were included. Patient's survival rates at 1 and 10 years were 97.6% (CI 95% 97.4–97.8) and 83.1% (CI 95% 81.9–84.3), respectively. The multivariate analysis showed that male patients, over 65 years old and underweight had an increased risk of death. Therapeutic regimens containing a bronchodilator in a free dose along with a fixed-dose combination of corticosteroid and bronchodilator seem to be a protective factor when compared to other regimens.

**Conclusion:**

Our findings contribute to the knowledge of COPD patients' profile, survival rate and related risk factors, providing new evidence that supports the debate about pharmacological therapy and healthcare of these patients.

## Introduction

Chronic obstructive pulmonary disease (COPD) is a highly prevalent disease with an increasing incidence among people over the age of 40. It currently affects ~10% of the world's population in this age group, increasing up to 33% among the female population over 70 years of age (Prince et al., 2015), and is the third leading cause of death worldwide (Celli and Wedzicha, 2019). Low- and middle-income countries (LMICs) are experiencing a disproportionate increase in non-communicable diseases (NCD), with COPD already being a serious public health issue (WHO, 2019). A recent meta-analysis by Cruz and Santos estimated the prevalence of COPD among adults aged ≥ 40 years in Brazil at 19% (CI 95% 14.0–24.0%) (Cruz and Santos, 2019).

The goal of treating COPD is to reduce mortality and the risk of hospitalization, to improve exercise tolerance and quality of life, and to control symptoms. Randomized clinical trials (RCTs) have documented that pharmacological treatment may be able to modify mortality rates, and reduce lung function decline (Celli et al., 2009; Scott et al., 2015; Bengtson et al., 2018). Overall, pharmacotherapy can affect exacerbation and hospitalization rates, the physical condition (Barnes et al., 2015), and the frequency and severity of exacerbations, which are closely related to increased risk of mortality, decline in lung function, and worsening of quality of life (Ho et al., 2014).

Medicines used in the pharmacological treatment of COPD includes beta2-agonists and anticholinergics. International guidelines for COPD recommend a “step-by-step” approach based on disease severity (GOLD, 2020). Therapy is initiated with short-acting bronchodilators, as needed, for symptom relief. For patients with persistent symptoms, the use of one or two classes of long-acting bronchodilators is recommended. Special concerns about inhaled glucocorticoids (ICS) safety in recent years, including an increased risk of pneumonia, have resulted in changes in pharmacotherapy in the past years, with the ICS/LABA combination now being recommended only for a restricted group of patients who show frequent exacerbations and a high eosinophil count, or a history/clinical record of associated asthma (Chalmers and Keir, 2017; Godman et al., 2020; GOLD, 2020).

Brazil has a public-funded National Health System, Sistema Único de Saúde (SUS), that provides fully subsidized access to prescribed medicines, including those for COPD. The national formulary includes long-(LABA) and short-acting beta-2-agonists (SABA), short-acting antimuscarinic agents (SAMA), and inhaled corticosteroids (ICS), with other medicines available in the market via private prescription or offered by State governments to their citizens. This is the case for long-acting antimuscarinic agents (LAMA), which are currently provided by 11 out of 27 States of Brazil. Prescription of COPD subsidized medicines should comply with Brazilian Clinical Protocol and Therapeutic Guidelines. Currently, the guideline recommends ICS for patients with severe COPD, with ≥ 2 exacerbations in the past year. However, there were recent modifications in COPD medicines listed in the national formulary and clinical recommendations; these will be implemented nationally in the near future (CONITEC/MH, 2019).

Most evidence based current Brazilian guidelines are from RCTs. While randomized clinical trials (RCTs) are designed to provide the best possible evidence on efficacy, different limitations inherent in the study design could impact the reliability and external validity of the results for the general population. For instance, the highly controlled environment and the patient selection criteria of RCTs used for registration purposes, designed to assure internal validity, may not fully reflect real-world clinical practice due to potentially older patients and with more health problems than seen in clinical trials (Malmström et al., 2013). Moreover, RCT results may not apply to the health context of different countries and communities around the world, and there could be issues of adherence to prescribed medication regimen in routine care. Consequently, especially in universal health care systems, it becomes fundamental to assess the effectiveness, safety, and value of funded technologies using real world data (Guerra-Júnior et al., 2017).

As previous studies have shown, the use of administrative database can provide valuable evidence for decision-making in healthcare (Liu et al., 2021). The Brazilian Federal government maintains several information systems that capture dispensing claims of medicines, notifiable diseases, hospitalisations and deaths. Previous efforts to link those data culminated in a large dataset that enables measurement of medicines exposure and outcomes at the person level for the entire Brazilian population (Guerra et al., 2018). These data are a valuable source for monitoring the uptake, pattens of use, and outcomes of subsidized medicines in Brazil. The evidence generated from these data have the potential to inform clinical practice and national guidelines.

In this context, we aimed to assess the survival of patients with COPD in Brazil and to identify risk factors associated with increased survival in an open cohort with 13 years of follow-up using real-world data. We focused on which treatments and regimens are associated with better outcomes among this population with currently only regional data available in Brazil (Pinto et al., 2019).

## Methods

### Study Design and Data Source

We conducted a retrospective cohort study of patients who underwent COPD treatment in SUS from 01/01/2000 to 12/31/2015. A National Database of Health centered on the individual was built through a deterministic-probabilistic record linkage of three administrative databases: the Outpatient Information System (SIA/SUS), the Hospital Information System (SIH/SUS), and the Mortality Information System (SIM). The construction and validation of this database have been described elsewhere (Guerra et al., 2018), and it has been used in previous studies published by our research group in Brazil (Lemos et al., 2018; Acurcio et al., 2020; Nascimento et al., 2020).

### Study Cohort

The time period of the cohort was determined based on the data available within the database.

Patients were included in the full cohort if they were over 40 years old and were dispensed the following medicines for COPD treatment: beclomethasone, budesonide, formoterol/budesonide, fenoterol, formoterol, salbutamol or salmeterol; for the following diagnosis [according to the tenth revision of the International Classification of Diseases (ICD-10)]: J44.0 J44.1, or J44.8. According to the guideline, the diagnosis should be confirmed by spirometry. The index date was defined as the first-dispensing date of the aforementioned medicines in the observation window, including their respective diagnosis.

In order to more accurately evaluate the effectiveness of treatment and to guarantee the assessment of patients who persisted in treatment for longer periods of time, patients with an index date after December 31, 2014, and patients on medicines for three months or less were excluded. We also matched patients 1:1 using the variables sex, age in years at the index date and type of regimen dispensed at the index date. When more than one patient met the matching criteria, pair allocation was randomly selected. We defined as fixed-dose combination (FDCs) medicines dispensed containing formoterol and budesonide in the same formulation. Medicines dispensed in non FDCs formulations were classified as free-dose regimens (FDS). Finally, patients who received both FDCs and FDS in their index date were classified as receiving mixed-dose regimens (MDR).

### Study Measurements

The event of interest for the survival analysis was death. All patients were followed from the index date until death or until December 2015 (right censoring), and loss of follow-up was defined as informative censoring.

Baseline characteristics were reported in a descriptive analysis of all variables according to data recorded at the index date. Explanatory variables included patients' sociodemographic characteristics at baseline. Weight and height information at baseline were used to calculate body mass index (BMI) according to WHO parameters (Liu et al., 2021). Other variables were previous diagnosis of asthma and lung cancer, therapeutic regimens and pharmacological class of medicines first-dispensed. First-dispensed medicines do not represent first-line treatment, as the data is not exclusive for naïve patients.

Hospitalization at any time during follow-up due to respiratory causes was grouped according to ICD-10 in six groups: hospitalization for COPD exacerbation, asthma, bronchitis or emphysema, respiratory infections except for pneumonia, pneumonia, and other respiratory problems (all respiratory diseases not included in the previous categories).

Comorbidity scores were calculated based on the Charlson Comorbidity Index (CCI), considered as a proxy of patient severity, using medical services records in the database from three years prior to the index date (Charlson et al., 1987; Quan et al., 2005). The higher the CCI, the greater the severity of the patient; low severity corresponds to a CCI between 0 and 1 and, and high severity to a CCI ≥2 (Liu et al., 2020). General patient frailty (frailty index) was calculated as the number of in-hospital days for any cause during the two years prior to the index date (Neovius et al., 2015).

### Statistical Analysis

Survival was assessed using the Kaplan Meier method, and the log-rank test was used to compare patients' baseline characteristics and therapeutic regimens. Factors influencing survival rates were assessed initially by univariate analysis. Clinically relevant variables previously demonstrated in the literature, such as being underweight or age, and those with a *p*-value <0.20 in the univariate analysis were included in the multivariable Cox proportional hazards model. Adjusted hazard ratios (HRs) and 95% Confidence Intervals (CI) were calculated in the multivariable model, and their suitability was assessed by residue analysis. Schoenfeld residuals were used to check the proportional hazards assumption (Schoenfeld, 1982).

The univariate and multivariate analysis were performed only for the matched cohort. A sensitivity analysis was performed to better understand which drugs inside the MDR category were related to better survival rates in the multivariate model.

Statistical analysis was performed using R version 4.0.2 R Foundation for Statistical Computing, considering a significance level of 5%.

### Ethics Statement

The research was approved by the Ethics Research Committee of the Federal University of Minas Gerais 165 (Report No. 16334413.9.0000.5149), and anonymity was maintained with regards to all patient data.

## Results

**Figure 1:**
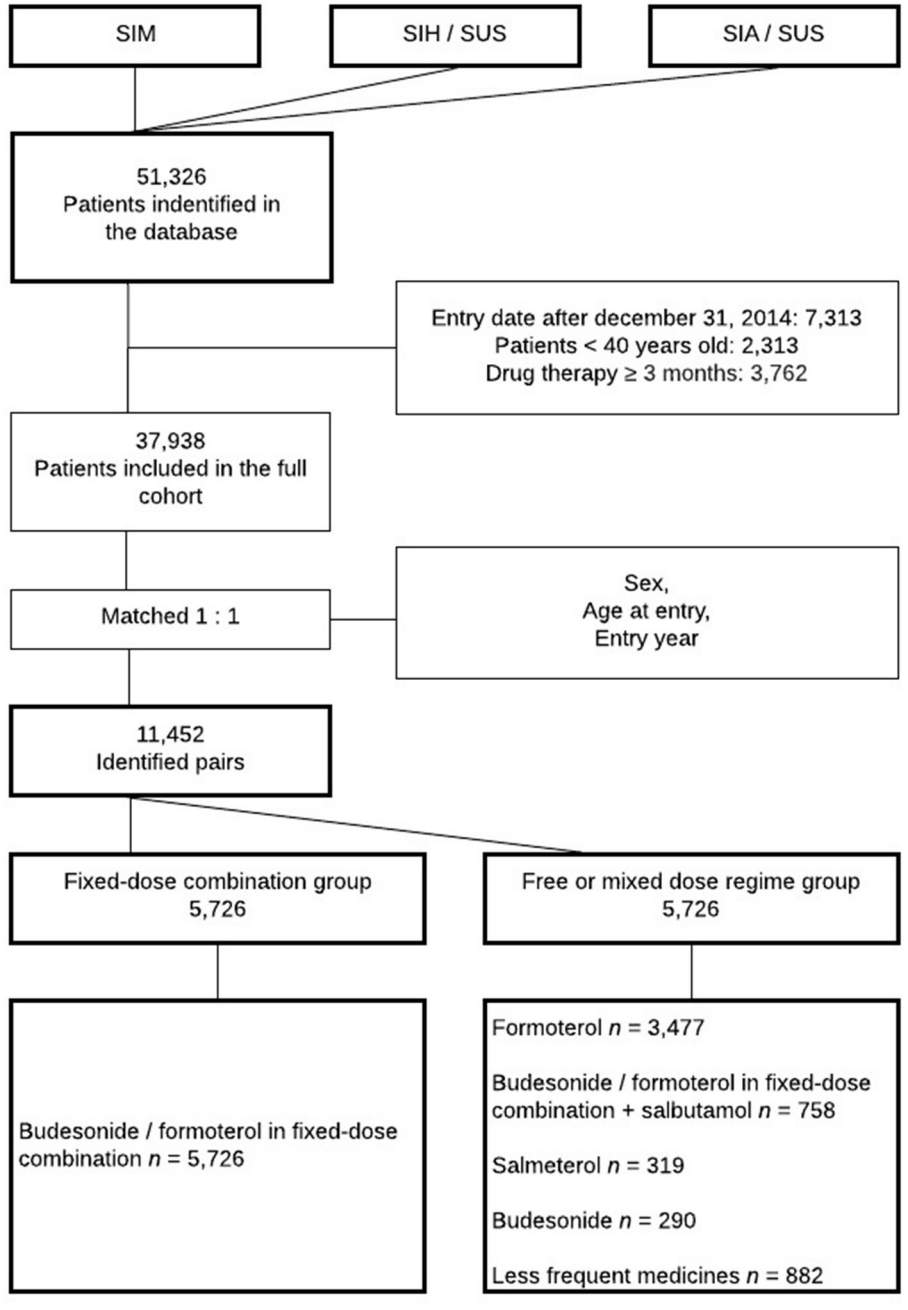
Cohort selection flowchart. SUS, Brazilian National Health System; SIM, Mortality Information System; SIH/SUS, Hospital Information System, SIA/SUS Outpatient Information System.

Thirty seven thousand and nine hundred and thirty eight patients were included, of which 11,452 were matched for treatment comparisons (Figure 1). The mean follow-up was 37.8 and 37.7 months, and mean age of 65.2 and 64.8 years for both cohorts, respectively. Most patients lived in the southeastern region of Brazil. Skin color data was not available for approximately half of the patients, with white skin color being the most common (33.3 for full cohort and 35.6% for matched cohort) among available data. Patients started to enter the cohort in 2003, and most of them (>76%) entered in recent years (2011–2015). The baseline characteristics of the patients are summarized in Table 1.

**Table 1:** Baseline characteristics of patients included in the Cohort from 2000 to 2015.

**Characteristic**		**Full cohort*****n*** **=** **37,938**		**Matched cohort*****n*** **=** **11,452**		**Fixed-dose combination*n* = 5,726**	**Free and mixed-dose regime*n* = 5,726**
		**n**	**%**	**n**	**%**	**%**	**%**
Sex	Female	18,831	49.6	5,152	45.0	22.5	22.5
	Male	19,107	50.4	6,300	55.0	27.5	27.5
Age group	40–45	1,411	3.7	420	3.67	1.8	1.8
	46–55	6,071	16.0	1,976	17.25	8.6	8.6
	56–65	11,517	30.4	3,576	31.23	15.6	15.6
	> 65	18,939	49.9	5,480	47.85	23.9	23.9
Skin color	White	12,706	33.5	4,077	35.6	16.1	19.5
	Brown	3,189	8.4	1,120	9.8	4.0	5.8
	Asian/yellow	1,497	3.9	405	3.5	2.2	1.3
	Black	807	2.1	281	2.5	0.9	1.5
	Indigenous	9	0.0	4	0.0	0.0	0.0
	Undeclared	19,730	52.0	5,565	48.6	26.7	21.9
Region of residence	Southeast	30,053	79.2	9,520	83.13	38.6	44.5
	South	4,316	11.4	1,016	8.87	6.3	2.5
	Northeast	2,344	6.2	563	4.92	3.4	1.5
	North	666	1.8	187	1.63	0.7	0.9
	Midwest	559	1.5	166	1.45	0.9	0.5
BMI	Underweight	1,793	4.7	519	4.53	2.3	2.2
	Normal weight	13,491	35.6	3,824	33.39	17.9	15.5
	Overweight	8,567	22.6	2,473	21.59	11.9	9.7
	Obesity	4,805	12.7	1,369	11.95	6.4	5.6
	Not recorded	9,282	24.5	3,267	28.53	11.5	17.0
Entry date (year)	2003 to 2006	2,187	5.8	802	7.0	3.5	3.5
	2007 to 2010	6,783	17.9	1,830	16.0	8.0	8.0
	2011 to 2014	28,968	76.4	8,820	77.0	38.5	38.5
Event	Censoring	35,868	94.5	10,839	94.7	47.1	47.5
	Death	2,070	5.5	613	5.4	2.9	2.5
Comorbidities and frailty	Median CCI (IQR)	2.0 (1.0, 1.0)		1.0 (1.0, 1.0)		1.0 (1.0, 1.0)	1.0 (1.0, 1.0)
	Median Frailty Index (IQR)	0.0 (0.0, 2.0)		0.0 (0.0, 2.0)		0.0 (0.0, 1.0)	0.0 (0.0, 2.0)
First-dispensed medicine	LABA + ICS	32,625	86.0	6,186	54.0	50.0	4.0
	LABA monotherapy	3,818	10.1	3,797	33.2	NA	33.2
	LABA + ICS + SABA	840	2.2	826	7.2	NA	7.2
	ICS monotherapy	397	1.0	389	3.4	NA	3.4
	SABA monotherapy	168	0.4	166	1.4	NA	1.4
	LABA + SABA	49	0.1	48	0.4	NA	0.4
	SABA + ICS	41	0.1	40	0.3	NA	0.3

*Age group: 40 years ≥ Adult <65 years, Elderly ≥65 years; BMI: underweight <18.5 kg/m^2^, 18.5 kg/m^2^ ≥ normal weight > 24.9 kg/m^2^, 25.0 kg/m^2^ ≥ overweight > 29.9 kg/m^2^, obesity ≥ 30.0 kg/m^2^; CCI, Charlson Comorbidity Index; Other RD, other respiratory diseases; ICS, inhaled corticosteroid; SABA, short-acting beta-2-agonist; LABA, long-acting beta-2-agonist; FDC, fixed-dose combination*.

### Survival Analysis

**Figure 2:**
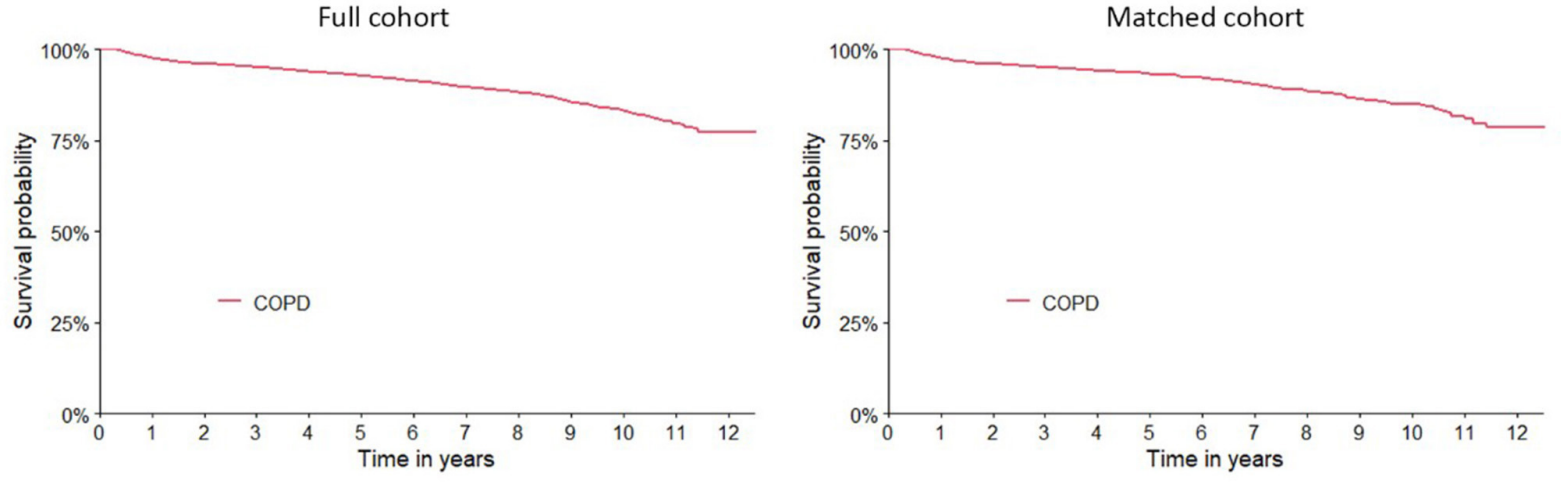
Kaplan-Meier survival curves for COPD patients from 2003 to 2015 included in full and matched cohort.

**Figure 3:**
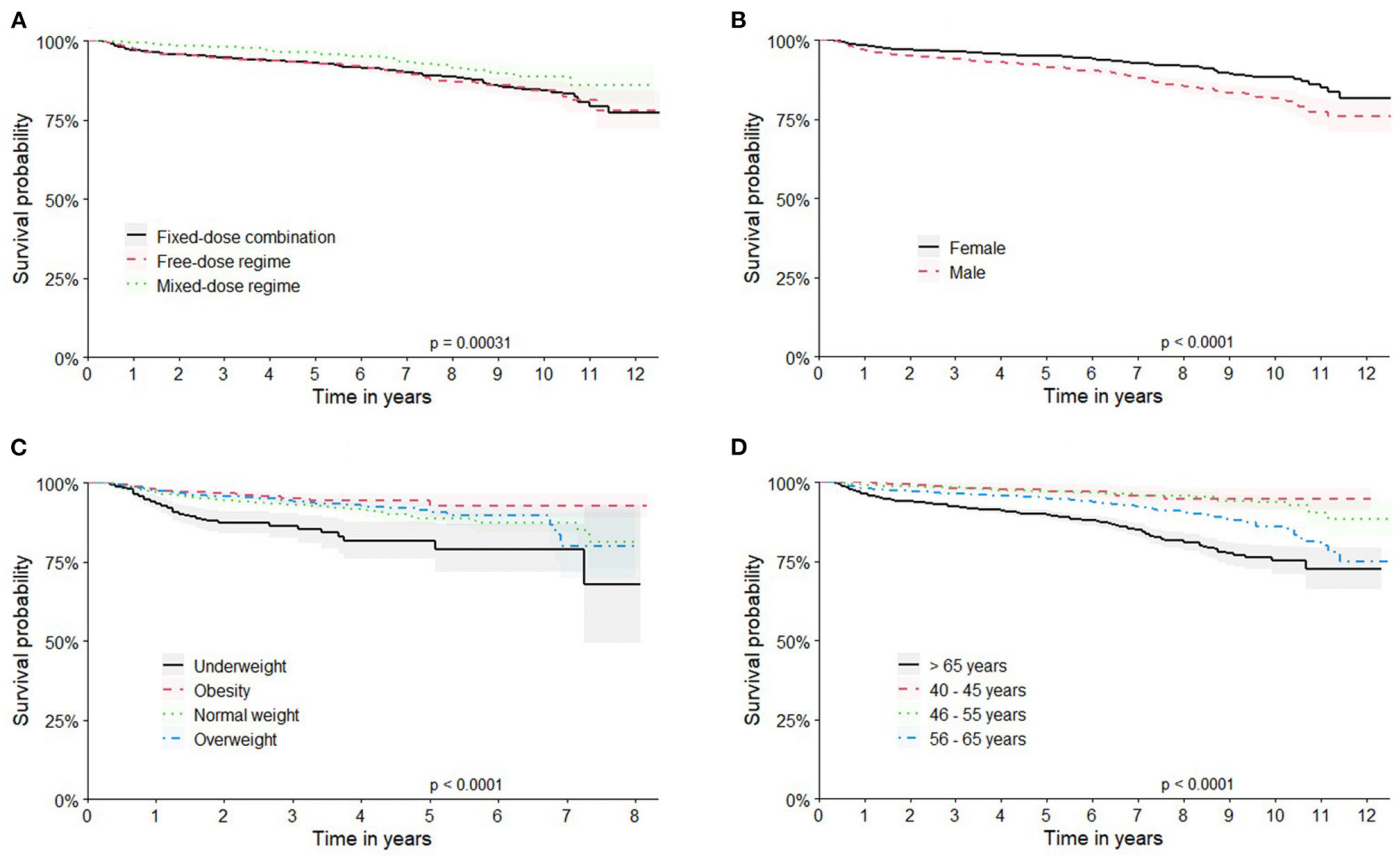
Kaplan-Meier curves for patient survival in the Matched cohort. Longitudinal survival by: **(A)** therapeutic regimens; **(B)** patient's sex; **(C)** Body Mass Index category; **(D)** age group. Survival rates were estimated by Kaplan-Meier methodology and compared by log rank test.

In the full cohort, a total of 2,070 deaths occurred (5.5%), and patients' survival rates at 1 and 10 years were 97.6% (CI 95% 97.4–97.8) and 83.1% (CI 95% 81.9–84.3), respectively (Supplementary Table 1). Similar results were obtained for the matched cohort (Figure 2). The survival curves categorized by the characteristics of the patients and therapeutic regimens for the matched cohort are shown in Figure 3. Elderly patients (> 65 years), male or underweight patients had significantly shorter survival rates than younger patients, females and normal weight, overweight and obesity patients. When assessed by therapeutic regimens, patients on MDR at baseline had better survival than patients on FDCs alone (HR 0.57, CI 95% 0.43–0.76).

### Univariate Analysis

The univariate analysis indicated a higher risk of death for men (HR 1.67 [CI 95%: 1.42–1.97]) and elderly patients (HR: 2.69 [CI 95% 2.27–3.18]). The BMI category underweight (HR 2.51 [CI 95% 1.89–3.32]) was also a risk factor for COPD survival. A lower risk was found for patients in the Southeast region (HR 0.74 [CI 95% 0.67–0.83]) (Table 2).

**Table 2:** Univariate analysis of baseline characteristics associated with survival in COPD in the matched cohort.

**Variable**	**HR (CI 95%)**	***p-value***
**Male sex**	1.67 (1.42–1.97)	<0.001
**Region of residence**		
Midwest	1.13 (0.56–2.27)	0.7
Northeast	1.69 (1.21–2.36)	0.004
North	0.67 (0.32–1.40)	0.3
Southeast	0.70 (0.57–0.86)	0.001
South	1.43 (1.10–1.86)	0.01
**Age group**		
Adult (40–64 years old)	1	
Elderly (65 years or older)	2.69 (2.27–3.18)	<0.001
**BMI**		
Underweight	2.51 (1.89–3.32)	<0.001
Normal weight	1.11 (0.91–1.35)	0.3
Overweight	0.80 (0.64–0.99)	0.014
Obesity	0.61 (0.45–0.83)	<0.001
**Skin color**		
Yellow (asian)	1.07 (0.67–1.70)	0.8
White	1.03 (0.81–1.31)	0.8
Brown	0.94 (0.70–1.25)	0.6
Black	1.00 (0.62–1.64)	1
**Entry date (additional year)**	1.63 (1.52–1.74)	<0.001
**Asthma diagnosis**	0.47 (0.40–0.56)	<0.001
**Lung cancer**	4.13 (2.98–5.71)	<0.001
**CCI**	1.19 (1.17–1.22)	<0.001
**Frailty index**	1.01 (1.00–1.01)	<0.001
**Hospitalization due to**		
Pneumonia	2.79 (2.30–3.39)	<0.001
Respiratory infections	4.69 (2.85–7.71)	<0.001
Bronchitis/emphysema	2.25 (1.42–3.55)	0.002
Cardiovascular events	2.74 (2.24–3.36)	<0.001
COPD exacerbations	2.79 (1.95–3.98)	<0.001
Asthma	1.48 (0.96–2.27)	0.09
Other RD	3.60 (2.66–4.87)	<0.001
Pharmacological class*		
ICS	0.59 (0.36–0.97)	0.02
LABA	1.39 (1.17–1.66)	<0.001
LABA + ICS	1.08 (0.92–1.26)	0.4
SABA	0.79 (0.42–1.48)	0.5
SABA + ICS	0.50 (0.12–2.00)	0.3
SABA + LABA + ICS	0.53 (0.38–0.73)	<0.001
Medicines*		
Budesonide	0.65 (0.36–1.18)	0.1
Formoterol	1.30 (1.09–1.56)	0.005
Formoterol/budesonide	1.13 (0.97–1.33)	0.1
Formoterol/budesonide + Salbutamol	0.56 (0.41–0.79)	<0.001
Salmeterol	1.78 (1.17–2.71)	0.01
Infrequent medicines	0.60 (0.45–0.81)	<0.001
Dispensed regime*		
FDC	1	
Free-dose regime	1.01 (0.86–1.20)	<0.001
Mixed-dose regime	0.57 (0.43–0.76)	
Dispensed regime detailed*		
FDC	1	<0.001
Corticosteroid + bronchodilators in free-dose	0.65 (0.39–1.08)	
Only bronchodilators in free-dose	1.14 (0.95–1.36)	
Only corticosteroids in free-dose	0.56 (0.34–0.92)	
FDC + corticosteroids and bronchodilators in free-dose	0.35 (0.09–1.40)	
FDC + a bronchodilator in free-dose	0.59 (0.43–0.81)	
FDC + a corticosteroid in free-dose	0.54 (0.28–1.04)	

^*^Of the first-dispensed medicines.

*Age group: 40 years ≥ Adult <65 years, Elderly ≥65 years; BMI: underweight <18.5 kg/m^2^, 18.5 kg/m^2^ ≥ normal weight > 24.9 kg/m^2^, 25.0 kg/m^2^ ≥ overweight > 29.9 kg/m^2^, obesity ≥ 30.0 kg/m^2^; CCI, Charlson Comorbidity Index; Other RD, other respiratory diseases; ICS, inhaled corticosteroid; SABA, short-acting beta-2-agonist; LABA, long-acting beta-2-agonist; FDC, fixed-dose combination*.

The analysis revealed that, when compared to FDC, the regimens that included corticosteroids in free-dose (HR 0.56 [CI 95%, 0.34–0.92]), or the MDR containing the FDC plus a bronchodilator in free-dose (HR 0.59 [CI 95% 0.43–0.81]) offered more protection to the patient.

### Multivariate Analysis

The multivariable analysis demonstrated an increased risk of death associated with male patients (HR 1.38; [CI 95%, 1.12–1.70]), patients over 65 years old, when compared to the other age groups, being underweight and a higher classification in the CCI. Moreover, having been hospitalized for cardiovascular events or pneumonia also demonstrated a strong risk relationship with survival. With respect to the regimes, the mixed-dose regime base on the association of FDC and a bronchodilator in a free-dose regimen tended to be a protective factor when compared to the FDC of formoterol/budesonide (Table 3).

**Table 3:** Estimated Hazard Ratio at a 95% Confidence Interval according to the Cox Proportional analysis model for the matched cohort from 2003 to 2015 (*n* =8,185).

**Variable**	**HR (CI 95%)**	***p***
Male sex	1.38 (1.12–1.70)	0.0025
Age >65 years	1	
56–65 years	0.52 (0.41–0.67)	<0.001
46–55 years	0.21 (0.13–0.33)	<0.001
36–45 years	0.22 (0.10–0.51)	<0.001
Underweight	1	
Obesity	0.40 (0.27–0.59)	<0.001
Normal weight	0.56 (0.41–0.75)	<0.001
Overweight	0.43 (0.31–0.59)	<0.001
ICC	1.14 (1.10–1.17)	<0.001
FDC	1	
Corticosteroid + bronchodilators in free-dose	0.79 (0.39–1.62)	0.5243
Only bronchodilators in free-dose	0.89 (0.71–1.11)	0.2969
Only corticosteroids in free-dose	0.54 (0.25–1.15)	0.1108
FDC + corticosteroids and bronchodilators	0.54 (0.08–3.87)	0.5397
in free-dose		
**FDC** **+** **a bronchodilator in free-dose**	**0.51 (0.32–0.80)**	**0.0033**
FDC + a corticosteroid in free-dose	0.82 (0.34–2.00)	0.6642
Hospitalization due to cardiovascular events	2.10 (1.59–2.77)	<0.001
Hospitalization due to pneumonia	2.61 (2.01–3.41)	<0.001
**Concordance** **=** **0.748** ***p*** ** = < 0.001**		

*Regimen that showed a protective factor when compared to FDC alone is highlighted in bold*.

Sensitivity analysis results: When compared to other medicines, the first-dispensing of FDC plus salbutamol—a short-acting bronchodilator—showed greater protective effect (HR 0.50; [CI 95%, 0.30–0.92]) (Supplementary Table 2). Analysis of the residues, according to Schoenfeld, demonstrated that both models present good adequacy.

## Discussion

This study evaluated a large national cohort of COPD patients in an LMIC, being a relevant source of evidence for LMIC settings. Survival and factors associated with medication use for COPD in LMICs are currently poorly understood. We present for the first-time data on outpatients receiving standard care provided by SUS. These data show the sociodemographic and clinical features of non-hospitalized COPD patients with spirometry diagnostic confirmation in a nationwide cohort. They also reflect the survival of patients with COPD and the associated risk factors in outpatients, which is quite important since most survival analysis of COPD comes from hospitalized patients.

Previously published studies in the literature on the survival of COPD patients primarily assessed data from hospitalized patients or specific subgroups—usually high-risk patients—with much lower survival rates, such as patients on long-term oxygen therapy (80.9 and 7.1%, respectively, in 1 and 10 years) (Foucher et al., 1998) or patients with a long history of smoking (survival of 16.0% in 10 years) (Miniati et al., 2014). Some studies have assessed the survival of COPD patients after hospitalization or admission to intensive care (Wildman et al., 2009; Lash et al., 2011). Therefore, there were substantial differences between our study population, study design and variables ascertained that are not reproducible with our data. Moreover, our study uses a robust database, with outpatient-level information and national-level data from a Universal Health System. In this sense, we are not aware of other studies developed with these configurations that can match our data for direct comparisons.

Our data corroborate studies of sex differences among COPD patients that showed better survival among women (Sunyer et al., 1998; Institute for Health Metrics Evaluation, 2017; Perez et al., 2020). Low BMI is recognized as a poor prognostic factor of COPD, affecting 25–40% of all patients. A combination of pathophysiological changes of malnutrition is also associated with higher mortality rates (Slinde et al., 2005; Vestbo et al., 2006; Itoh et al., 2013). Malnutrition is a well-recognized risk factor for mortality among COPD patients at all stages of the disease, and BMI is one of the four criteria of the BODE (body mass index, airflow obstruction, dyspnea, and exercise capacity) index for mortality (Landbo et al., 1999; Celli et al., 2004). Hence, the importance of nutritional care programs needs to be emphasized.

A systematic review and meta-analysis showed that among 14 different pharmacological treatments for COPD, only indacaterol, an ultra-long-acting beta-2-agonist, and the combination of salmeterol and fluticasone propionate—an ICS—were associated with a reduction in the all-cause mortality risk (Scott et al., 2015). Although salmeterol-fluticasone has been available in some states in Brazil, neither medications are available on a nationwide basis, which is restricted to formoterol/budesonide (ICS/LABA). However, ICS/LABA combination is now only recommended for a restricted group of patients (Chalmers and Keir, 2017; Godman et al., 2020; GOLD, 2020). Since 2016, the addition of ICS to long-acting bronchodilators basal treatment has been recommended for patients with frequent exacerbations (Brozek et al., 2019; Tsiligianni et al., 2019), which means that <20% of COPD patients would have an indication for treatment with ICS. On the other hand, GOLD 2017 states that ICS combined with LABA is more effective than the individual components in improving lung function (Barnes, 2017). The slow change in strategy probably explains why studies show that more than 70% of diagnosed patients are typically being treated with an ICS, and ~50% of newly diagnosed patients are started with an LABA/ICS FDC (Costa, 2017). Our study showed that this proportion is even higher, which may have occurred because of the narrow treatment options available in SUS. Another important consideration comes from the PLATINO study, that showed under diagnosis is the hallmark of COPD in Brazil (Moreira et al., 2014), and the less severe forms—the so-called GOLD A and B with no indication of ICS, are frequently not diagnosed. Certainly, our diagnosed cases were more frequent in categories with ICS indication.

Since the indication of ICS is restricted to a specific group of patients due to its safety profile and risk/benefit ratio, such associations in FDC may become questionable. The reasons justifying FDCs are controversial. The main reasons for FDCs are patient convenience and treatment adherence. Some studies have assessed the effectiveness and cost-effectiveness of LABA/ICS FDC, but overtreatment, effectiveness peaks at different times, lack of titration and potential to increase polypharmacy are still important issues regarding FDCs in general (Godman et al., 2020). An FDC with an ICS has the potential to lead to overmedication with steroids, since COPD treatment is indicated according to disease stage, and can change according to the frequency of exacerbations and symptoms. It may be necessary for the patient to use a bronchodilator as a rescue medication when symptoms increase and with only a bronchodilator and corticosteroid FDC prescribed, patients may exceed the optimal dosing of ICS, increasing the risk of adverse events (Godman et al., 2020). Supporting this hypothesis, the final model proposed in our study showed better survival rates for patients who started the therapy with an short-acting bronchodilator in free dosing regimens along with the FDC, after controlling for other variables (Jardim and Nascimento, 2006).

SABAs are typically used as rescue medications because they provide fast symptom relief and are recommended as an initial pharmacological treatment for all patients with COPD (GOLD, 2020). However, short-acting bronchodilators are also available in primary care in SUS, and tiotropium is available only in a few States. For these reasons, the total information about their use was not available in the database used in this study, which did not collect data from primary care pharmacies. This represents a limitation of our findings.

Our study has several other limitations related to the data availability and the use of administrative data for research. We only had data available until 2015 given the creation of this unique dataset requires complex linkage methods that hampers its frequent update. It is not possible to ascertain how results would change in more recent years considering the availability of new medicines in the health care system. Moreover, we did not capture data on private prescriptions or subsidized medicines dispensed by other programs—such as tiotropium bromide and ipratropium bromide. This may have impacted our study population selection and introduced residual confounding in the analysis.

Another limitation refers to the quality of data used, that depends on the registration and feeding process of the secondary information into the original database. Incorrect or incomplete data—an inherent limitation of secondary databases—may underestimate or overestimate the analysis results, particularly for clinical variables and non-mandatory fields (e.g., weight and race).

However, in the absence of more detailed clinical records, the use of variables such as CCI and selected causes of hospitalization made it possible to stratify patients' severity as a proxy in our analysis. Finally, we did not control the analysis by changes in pharmacotherapy or treatment adherence that are likely to influence treatment outcomes. Despite these limitations, we believe our findings are robust enough to add to the data on the management of COPD in LMICs and can help in providing future directions.

## Data Availability Statement

The datasets presented in this article are not readily available because the administrative databases used were provided by the Ministry of Health to the University in order to carry out epidemiological and research to guide decision making in health and for this will not be publicized. Requests to access the datasets should be directed to DataSUS, datasus@saude.gov.br.

## Author Contributions

LG, AG, FA, and JÁ-T contributed to conception and design of the study. LG and AG performed the statistical analysis. All authors contributed to the first draft of the manuscript till the manuscript revision, read, and approved the submitted version.

## Funding

This research is supported by the NHMRC Centre of Research Excellence in Medicines Intelligence (ID: 1196900) and by the Coordination of Superior Level Staff Improvement (CAPES).

## Conflict of Interest

In 2020, the Centre for Big Data Research in Health, University of New South Wales Sydney has received funding from AbbVie Australia to conduct post-market surveillance research. AbbVie did not have any knowledge of, or involvement in, the current study. The authors declare that the research was conducted in the absence of any commercial or financial relationships that could be construed as a potential conflict of interest.

## Publisher's Note

All claims expressed in this article are solely those of the authors and do not necessarily represent those of their affiliated organizations, or those of the publisher, the editors and the reviewers. Any product that may be evaluated in this article, or claim that may be made by its manufacturer, is not guaranteed or endorsed by the publisher.

## References

[B1] Acurcio F. de A. Guerra Junior A. A. da Silva M. R. R. Pereira R. G. Godman B. Bennie M.. (2020). Comparative persistence of anti-tumor necrosis factor therapy in ankylosing spondylitis patients: a multicenter international study. Curr. Med. Res. Opin. 36, 677–686. 10.1080/03007995.2020.1722945 31990224

[B2] Barnes P. J.. (2017). GOLD 2017. Chest 151, 245–246. 10.1016/j.chest.2016.11.042 28183480

[B3] Barnes P. J. Burney P. G. J. Silverman E. K. Celli B. R. Vestbo J. Wedzicha J. A.. (2015). Chronic obstructive pulmonary disease. Nat. Rev. Dis. Prim. 1, 1–22. 10.1038/nrdp.2015.76 27189863

[B4] Bengtson L. G. S. DePietro M. McPheeters J. Fox K. M. (2018). Real-world outcomes in patients with chronic obstructive pulmonary disease initiating long-acting mono bronchodilator therapy. Ther. Adv. Vaccines 12, 1–14. 10.1177/1753466618772750 29737943 PMC5961922

[B5] Brozek G. M. Nowak M. Zejda J. E. Jankowski M. Lawson J. Pierzchała W. (2019). Consequences of changing the GOLD Reports (2007–2011–2017) on the treatment regimen of patients with COPD. COPD J. Chronic Obstr. Pulm. Dis. 16, 126–132. 10.1080/15412555.2019.1615872 31161814

[B6] Celli B. Decramer M. Kesten S. Liu D. Mehra S. Tashkin D. P. (2009). Mortality in the 4-year trial of tiotropium (UPLIFT) in patients with chronic obstructive pulmonary disease. Am. J. Respir. Crit. Care Med. 180, 948–955. 10.1164/rccm.200906-0876OC 19729663

[B7] Celli B. R. Cote C. G. Marin J. M. Casanova C. Montes de Oca M. Mendez R. A.. (2004). The body-mass index, airflow obstruction, dyspnea, and exercise capacity index in chronic obstructive pulmonary disease. N. Engl. J. Med. 350, 1005–1012. 10.1056/NEJMoa021322 14999112

[B8] Celli B. R. Wedzicha J. A. (2019). Update on clinical aspects of chronic obstructive pulmonary disease. N. Engl. J. Med. 381, 1257–1266. 10.1056/NEJMra1900500 31553837

[B9] Chalmers J. D. Keir H. R. (2017). 10 years since TORCH: shining a new light on the risks of inhaled corticosteroids in COPD. Eur. Respir. J. 50:1701582. 10.1183/13993003.01582-2017 28931673

[B10] Charlson M. E. Pompei P. Ales K. L. MacKenzie C. R. (1987). A new method of classifying prognostic comorbidity in longitudinal studies: development and validation. J. Chronic Dis. 40, 373–383. 10.1016/0021-9681(87)90171-8 3558716

[B11] CONITEC/MH (2019). Protocolos e Diretrizes do Ministério da Saúde [Internet]. Available online at: http://conitec.gov.br/pcdt-em-elaboracao

[B12] Costa C. H. da, and Rufino, R.. (2017). Quando devemos usar CI na DPOC?/When should we use IC in COPD? Pulmäo RJ 26, 15–18. https://pesquisa.bvsalud.org/gim/resource/en/biblio-883592

[B13] Cruz M. Santos M. (2019). Epidemiology of chronic obstructive pulmonary disease in Brazil: a systematic review and meta-analysis. Eur. Resp. J. 54:PA3319. 10.1183/13993003.congress-2019.PA3319 33175061

[B14] Foucher P. Baudouin N. Merati M. Pitard A. Bonniaud P. Reybet-Degat O.. (1998). Relative survival analysis of 252 patients with COPD receiving long- term oxygen therapy. Chest 113, 1580–1587. 10.1378/chest.113.6.1580 9631797

[B15] Godman B. Alrasheedy A. A. D Leong T. (2020). Fixed dose drug combinations–are they pharmacoeconomically sound? Findings and implications especially for lower- and middle-income countries. Expert Rev. Pharmacoecon. Outcomes Res. 20, 1–26. 10.1080/14737167.2020.1734456 32237953

[B16] GOLD (2020). Global Strategy for Diagnosis, Management and Prevention of COPD. The Global Initiative for Chronic Obstructive Lung Diseases (GOLD). Available online at: https://goldcopd.org/gold-reports/ (accessed April 13, 2021).

[B17] Guerra A. A. Jr, Pereira, R. G. Gurgel E. I. Cherchiglia M. Dias L. V. Ávila J.. (2018). Building the National Database of Health Centred on the individual: administrative and epidemiological record linkage - Brazil, 2000-2015. Int. J. Popul. Data Sci. 3:20. 10.23889/ijpds.v3i1.446 34095519 PMC8142958

[B18] Guerra-Júnior A. A. Pires De Lemos L. L. Godman B. Bennie M. Osorio-De-Castro C. G. S. Alvares J.. (2017). Health technology performance assessment: real-world evidence for public healthcare sustainability. Int. J. Technol. Assess. Health Care 33, 279–287. 10.1017/S0266462317000423 28641588

[B19] Ho T. W. Tsai Y. J. Ruan S. Y. Huang C. T. Lai F. Yu C. J. (2014). In-hospital and one-year mortality and their predictors in patients hospitalized for first-ever chronic obstructive pulmonary disease exacerbations: a nationwide population-based study. PLoS One 9:e0114866. 10.1371/journal.pone.0114866 25490399 PMC4260959

[B20] Institute for Health Metrics Evaluation (2017). Global Burden of Diseases (GBD). Available online at: https://vizhub.healthdata.org/gbd-compare/

[B21] Itoh M. Tsuji T. Nemoto K. Nakamura H. Aoshiba K. (2013). Undernutrition in patients with COPD and its treatment. Nutrients 5, 1316–1335. 10.3390/nu5041316 23598440 PMC3705350

[B22] Jardim J. R. Nascimento O. A. (2006). Impacto e Tratamento da Doença Pulmonar Obstrutiva Crônica (DPOC) no Brasil. Rev Racine. 32–47. 10.1590/S1806-37132006000800007

[B23] Landbo C. Prescott E. Lange P. Vestbo J. Almdal T. P. (1999). Prognostic value of nutritional status in chronic obstructive pulmonary disease. Am. J. Respir. Crit. Care Med. 160, 1856–1861. 10.1164/ajrccm.160.6.9902115 10588597

[B24] Lash T. L. Johansen M. B. Christensen S. Baron J. A. Rothman K. J. Hansen J. G.. (2011). Hospitalization rates and survival associated with COPD: a nationwide danish cohort study. Lung 189, 27–35. 10.1007/s00408-010-9274-z 21170722

[B25] Lemos L. L. P. de, Guerra Júnior, A. A. Santos M. Magliano C. Diniz I. Souza K.. (2018). The assessment for disinvestment of intramuscular interferon beta for relapsing-remitting multiple sclerosis in Brazil. Pharmacoeconomics 36, 161–173. 10.1007/s40273-017-0579-0 29139001 PMC5805817

[B26] Liu H. Wu X. Cao J. Jiao J. Zhu C. Song B.. (2020). Effect of comorbidity assessed by the charlson comorbidity index on the length of stay and mortality among immobile hemorrhagic stroke patients younger than 50 years. Front Neurol. 11:487. 10.3389/fneur.2020.00487 32625159 PMC7314940

[B27] Liu M. Wang M. Li S. (2021). Prognostic factors of survival in pancreatic cancer metastasis to liver at different ages of diagnosis: a SEER population-based cohort study. Front. Big Data 4:654972. 10.3389/fdata.2021.654972 34651122 PMC8507850

[B28] Malmström R. E. Godman B. B. Diogene E. Baumgärte C. Bennie M. Bishop I.. (2013). Dabigatran - A case history demonstrating the need for comprehensive approaches to optimize the use of new drugs. Front. Pharmacol. 4:39. 10.3389/fphar.2013.00039 23717279 PMC3653065

[B29] Miniati M. Monti S. Pavlickova I. Bottai M. (2014). Survival in COPD: impact of lung dysfunction and comorbidities. Med (United States) 93, 1–9. 10.1097/MD.0000000000000076 25211048 PMC4616266

[B30] Moreira G. L. Manzano B. M. Gazzotti M. R. Nascimento O. A. Perez-Padilla R. Menezes A. M. B.. (2014). PLATINO, a nine-year follow-up study of COPD in the city of São Paulo, Brazil: the problem of underdiagnosis. J. Bras. Pneumol. 40, 30–37. 10.1590/S1806-37132014000100005 24626267 PMC4075910

[B31] Nascimento J. H. P. Gomes B. F. de O. Carmo P. R. do, Petriz, J. L. F. Rizk S. I. Costa I. B. S. da S.. (2020). COVID-19 e Estado de Hipercoagulabilidade: Uma Nova Perspectiva Terapêutica. Arq. Bras. Cardiol. 114, 829–833. 10.36660/abc.20200308 32491074 PMC8386998

[B32] Neovius M. Arkema E. V. Olsson H. Eriksson J. K. Kristensen L. E. Simard J. F.. (2015). Drug survival on TNF inhibitors in patients with rheumatoid arthritis comparison of adalimumab, etanercept and infliximab. Ann. Rheum. Dis. 74, 354–360. 10.1136/annrheumdis-2013-204128 24285495 PMC4316855

[B33] Perez T. A. Castillo E. G. Ancochea J. Pastor Sanz M. T. Almagro P. Martínez-Camblor P.. (2020). Sex differences between women and men with COPD: a new analysis of the 3CIA study. Respir. Med. 171:106105. 10.1016/j.rmed.2020.106105 32858497

[B34] Pinto C. R. Lemos A. C. M. Assunção-Costa L. de Alcântara A. T. Yamamura L. L. L. Souza G. S.. (2019). Management of COPD within the Brazilian unified health care system in the state of bahia: an analysis of real-life medication use patterns. J. Bras. Pneumol. 45, 1–8. 10.1590/1806-3713/e20170194 30758425 PMC6534407

[B35] Prince M. J. Wu F. Guo Y. Gutierrez Robledo L. M. O'Donnell M. Sullivan R.. (2015). The burden of disease in older people and implications for health policy and practice. Lancet [Internet] 385, 549–562. 10.1016/S0140-6736(14)61347-7 25468153

[B36] Quan H. Sundararajan V. Halfon P. Fong A. Burnand B. Luthi J. C.. (2005). Coding algorithms for defining comorbidities in ICD-9-CM and ICD-10 administrative data. Med. Care 43, 1130–1139. 10.1097/01.mlr.0000182534.19832.83 16224307

[B37] Schoenfeld D.. (1982). Partial residuals for the proportional hazards regression model. Biometrika 69, 239–241. 10.1093/biomet/69.1.239

[B38] Scott D. A. Woods B. Thompson J. C. Clark J. F. Hawkins N. Chambers M.. (2015). Mortality and drug therapy in patients with chronic obstructive pulmonary disease: A network meta-analysis. BMC Pulm. Med. 15, 1–13. 10.1186/s12890-015-0138-4 26559138 PMC4642642

[B39] Slinde F. Grönberg A. M. Engström C. P. Rossander-Hulthén L. Larsson S. (2005). Body composition by bioelectrical impedance predicts mortality in chronic obstructive pulmonary disease patients. Respir Med. 99, 1004–1009. 10.1016/j.rmed.2004.09.024 15950141

[B40] Sunyer J. Antó J. M. Mcfarlane D. Domingo A. Tobías A. Barceló M. A.. (1998). Sex differences in mortality of people who visited emergency rooms for asthma and chronic obstructive pulmonary disease. Am. J. Respir. Crit. Care Med. 158, 851–856. 10.1164/ajrccm.158.3.9801093 9731016

[B41] Tsiligianni I. Kampouraki M. Ierodiakonou D. Poulonirakis I. Papadokostakis P. Lintovoi E.. (2019). COPD patients' characteristics, usual care, and adherence to guidelines: the Greek UNLOCK study. Int. J. COPD 14, 547–556. 10.2147/COPD.S185362 30880944 PMC6402614

[B42] Vestbo J. Prescott E. Almdal T. Dahl M. Nordestgaard B. G. Andersen T.. (2006). Body mass, fat-free body mass, and prognosis in patients with chronic obstructive pulmonary disease from a random population sample. Am. J. Respir. Crit. Care Med. 173, 79–83. 10.1164/rccm.200506-969OC 16368793

[B43] WHO (2019). Available online at: https://www.who.int/news-room/fact-sheets/detail/noncommunicable-diseases (accessed April 13, 2021).

[B44] Wildman M. J. Sanderson C. F. B. Groves J. Reeves B. C. Ayres J. G. Harrison D.. (2009). Survival and quality of life for patients with COPD or asthma admitted to intensive care in a UK multicentre cohort: the COPD and Asthma Outcome Study (CAOS). Thorax 64, 128–132. 10.1136/thx.2007.091249 18852157

