# Supplementary material for: Survival Analysis of COPD Patients in a 13-Year Nationwide Cohort Study of the Brazilian National Health System
Source: Front Big Data. 2022 Feb 7;4:788268. doi: 10.3389/fdata.2021.788268 (PMC8859158; doi:10.3389/fdata.2021.788268)
Supplement: Supplementary file 1 [file Table_1.DOCX]

Supplementary Material

**Appendix Table A1. Annual survival rates of the study patients in the full cohort and matched cohort according to the dose regime group.**

| **Follow-Up Year** | **Survival Rates (CI 95%)** | | |
| --- | --- | --- | --- |
|  | **Full cohort**  ***n* = 37,938** | **Fixed-dose regime group *n* = 5,726** | **Mixed and free dose group *n* = 5,726** |
| 1st | 97.6 (97.4 – 97.8) | 97.1 (96.7 – 97.6) | 97.9 (97.5 – 98.3) |
| 2nd | 96.1 (95.9 – 96.3) | 95.7 (95.2 – 96.3) | 96.3 (95.8 – 96.9) |
| 3rd | 95.2 (94.9 – 95.4) | 94.8 (94.1 – 95.5) | 95.4 (94.7 – 96.0) |
| 4th | 93.9 (93.6 – 94.2) | 93.8 (93.0 – 94.7) | 94.5 (93.7 – 95.3) |
| 5th | 92.7 (92.3 – 93.1) | 93.0 (92.1 – 94.0) | 93.5 (92.6 – 94.4) |
| 6th | 91.4 (91.0 – 91.9) | 91.6 (90.4 – 92.7) | 92.7 (91.7 – 93.8) |
| 7th | 89.6 (89.0 – 90.2) | 90.0 (88.6 – 91.4) | 90.7 (89.4 – 92.1) |
| 8th | 88.1 (87.4 – 88.8) | 88.7 (87.1 – 90.3) | 88.3 (86.5 – 90.1) |
| 9th | 85.5 (84.6 – 86.4) | 85.7 (83.5 – 87.9) | 86.9 (84.9 – 89.0) |
| 10th | 83.1 (81.9 – 84.3) | 84.3 (81.8 – 86.9) | 85.6 (83.2 – 88.0) |
| 11th | 79.6 (77.8 – 81.4) | 79.5 (75.0 – 84.2) | 82.9 (79.4 – 86.4) |

**Appendix Table A2. Estimated Hazard Ratio at a 95% Confidence Interval according to the Cox Proportional analysis model (n =8,185)**

| **Variable** | **HR (IC 95%)** | ***p*** |
| --- | --- | --- |
| Male sex | 1.37 (1.12 - 1.70) | 0.0027 |
| Age >65 years | 1 |  |
| 56 - 65 years | 0.52 (0.41 - 0.67) | <0.001 |
| 46 - 55 years | 0.21 (0.13 - 0.33) | <0.001 |
| 36 - 45 years | 0.22 (0.09 - 0.50) | <0.001 |
| Underweight | 1 |  |
| Obesity | 0.40 (0.27 - 0.59) | <0.001 |
| Normal weight | 0.55 (0.41 - 0.75) | <0.001 |
| Overweight | 0.43 (0.31 - 0.59) | <0.001 |
| ICC | 1.14 (1.10 - 1.17) | <0.001 |
| FDC + salbutamol in free dose | 0.50 (0.30 - 0.92) | 0.0058 |
| Hospitalization due to cardiovascular events | 2.08 (1.57 - 2.75) | <0.001 |
| Hospitalization due to pneumonia | 2.63 (2.02 - 3.42) | <0.001 |
| **Concordance= 0.746 p=<0.001** |  |  |
